# Physical performance on the early 6-minute walk test in coronary artery bypass grafting patients during inpatient cardiac rehabilitation

**DOI:** 10.3389/fcvm.2025.1580442

**Published:** 2025-07-31

**Authors:** Yao Wu, Bao Ren, Jing Li, Liqun Chi, Ping Li, Jiahui Wu

**Affiliations:** ^1^ Department of Cardiac Rehabilitation Center, Beijing Anzhen Hospital, Capital Medical University, Beijing, China; ^2^ Department of Minimally Invasive Surgery Center, Beijing Anzhen Hospital, Capital Medical University, Beijing, China

**Keywords:** CABG, cardiac rehabilitation, coronary artery disease, physical performance, 6MWT

## Abstract

**Background:**

The 6 min walk test (6MWT) is widely used to evaluate functional exercise capacity, therapeutic effects, and prognosis in patients with cardiopulmonary diseases. However, its application in phase I cardiac rehabilitation (CR) after coronary artery bypass grafting (CABG) remains under explored. This study investigates the physical performance of CABG patients during the early 6MWT and analyzes factors influencing their walking performance during inpatient CR.

**Methods:**

This retrospective study included 360 patients who underwent isolated off-pump coronary artery bypass (OPCAB, *n* = 240) or minimally invasive direct coronary artery bypass (MIDCAB, *n* = 120) surgery. Participants, with a median age of 63 years (range: 36–80), comprised 285 males and 75 females. The study was conducted from November 22, 2023, to December 25, 2024. It specifically included patients who completed the 6MWT within 5 ± 2 days during phase I CR post-surgery.

**Results:**

The median 6 min walk distance (6MWD) was 183 ± 125 meters (33 ± 20% of predicted). Notably, the walking distances were significantly shorter in OPCAB patients than MIDCAB patients (175 ± 125 vs. 200 ± 134 meters, *Z* = −3.426, *P* = 0.001), in older patients than younger patients (*H* = 20.489, *P* < 0.001) and in females than males (150 ± 84 vs. 200 ± 100 meters, *Z* = −5.919, *P* < 0.001). Univariate analysis showed 6MWD significantly correlated with height, weight, body mass index (BMI), diastolic blood pressure (DBP) and peripheral oxygen saturation (SpO2) before the test, as well as heart rate (HR), systolic blood pressure (SBP), DBP, SpO2, and respiratory rate (RR) after the test, and the mean values of HR and RR during the test. The stepwise multivariate regression analysis identified that gender, type of surgery, HR and RR at the end of the test, and DBP before the test were independent predictors of the 6MWD.

**Conclusion:**

This study is the first to describe early 6MWD in CABG patients during phase I CR. The 6MWT is feasible and well-tolerated in adults and older patients shortly after CABG. The findings provide valuable insights into factors affecting walking distance, aiding clinical assessment and informing phase II CR program development post-discharge.

## 1 Introduction

Cardiovascular diseases, which encompass a range of conditions affecting the heart and blood vessels, including coronary artery disease, cerebrovascular disease, rheumatic heart disease, and others, are the leading cause of death globally (1). Coronary artery bypass grafting (CABG) is an effective and established treatment for clinically significant coronary artery disease. It can effectively restore coronary blood flow, improve myocardial ischemia, and enhance cardiac function (2). Functional recovery post-CABG varies by surgical approach (OPCAB vs. MIDCAB), with minimally invasive techniques potentially facilitating earlier mobilization due to reduced trauma (3). Early assessment of physical capacity is critical for developing rehabilitation exercise prescription (4).

The 6 min walk test (6MWT) is a safe, simple, well-tolerated, and reliable tool for assessing the physical performance, the effects of the therapy and prognostic stratification of patients with severe cardiopulmonary disease (5–8). Early after cardiac surgery the estimated physical functional capacity of patients can be affected by perioperative factors, including prolonged bed rest, pain, anaemia, and restrictive respiratory patterns (9). Currently it is not known whether the distance walked during the 6MWT early after CABG surgery is associated with clinical and demographic factors. Therefore, the quantification of the relationship between the 6MWT distance and several clinical and demographic factors for patients who have undergone CBAG surgery is clinically important and relevant. Furthermore, the 6MWT serves as an indicator of phase I cardiac rehabilitation (CR) effectiveness and guides exercise prescription for phase II rehabilitation (10, 11). However, few studies have evaluated 6MWT performance during early inpatient CR after isolated CABG, particularly regarding the impact of surgical techniques (12). Thus, this study aimed to assess the physical performance of CABG patients in the early stages of the 6MWT during inpatient CR program, analyze the influence of various relevant clinical and demographic factors on the walking performance of these patients, and provide effective recommendations for feasibility of 6MWT as a measure of postoperative CR program after CABG.

## 2 Materials and methods

### 2.1 Study population

Subjects were enrolled among 360 inpatients from November 22, 2023 to December 25, 2024 for the phase Ⅰ cardiac rehabilitation program soon after either isolated off-pump coronary artery bypass (OPCAB) (*n* = 240) or isolated minimally invasive direct coronary artery bypass (MIDCAB) (*n* = 120) surgery. Patients requiring concomitant procedures were excluded. After returning to the ward from the intensive care unit after surgery, all participants initiated the phase Ⅰ CR program. The standardized phase Ⅰ CR program for patients who underwent CABG surgery should be begun as soon as possible, which included respiratory and cough training, upper and lower limb exercises, transfer training, standing training and graded walking exercise (13). Exercise training was conducted once a day and was supervised by members of the cardiac rehabilitation staff. Within the median of 5 ± 2 days after surgery, all subjects performed the 6MWT following the phase Ⅰ CR program and prior to hospital discharge according to the American Thoracic Society protocol, with electrocardiographic monitoring by telemetry. Their medical records were reviewed and assessed by two independent researchers. The study was approved by the hospital's ethical committee and informed consent was obtained from all study participants (KS2024003).

### 2.2 6-min walking test (6MWT)

According to the guidelines of the American Thoracic Society, the 6MWT was performed using a smart six minute walking test and analysis system (Wocaring Medical Equipment Co., Ltd.) (8). Patients were instructed to walk the greatest possible distance along a 25-meter straight, flat hospital corridor within 6 min (8, 14, 15). Prior to and following the 6MWT, measurements were taken of blood pressure (BP). During the test, patients wore intelligent monitoring devices (YK-2020A, Wocaring Medical Equipment Co., Ltd.) to record real-time changes in physiological indicators, including electrocardiography (ECG), heart rate (HR), respiratory rate (RR), and peripheral oxygen saturation (SpO_2_). A professional rehabilitation therapist accompanied the patients to ensure their safety without encouragement. The test was symptom-limited, and it was immediately discontinued if SpO_2_ fell below 85%, if ECG monitoring indicates severe arrhythmia, or if the patient experienced exhaustion, angina, severe dyspnoea, dizziness, diaphoresis, or intolerable leg cramps. The results of the 6MWT were given as absolute value in meters and as a percentage of the predicted value, taking into account anthropometric variables (age, sex, weight and height) according to the reference equation proposed by Enright and Scherril in healthy subjects (14). The metabolic equivalent (METs) of the patient was automatically calculated based on the 6MWT results and recorded. The oral informed consent was obtained from all participants and approved by the hospital's ethics committee (KS2024003).

### 2.3 Statistical analysis

We collected data on demographic variables such as gender and age, as well as clinical variables including height, weight, body mass index (BMI), type of surgery, time from surgery to the 6MWT, 6MWD, 6MWD as a percentage of predicted, METs, HR, systolic blood pressure (SBP), diastolic blood pressure (DBP), SpO_2_, and RR.

All statistical analyses were conducted using Statistical Product and Service Solutions (version 26.0 Inc., Chicago, IL, USA) and Microsoft Excel (Microsoft Corporation, Redmond, WA, USA). Data were presented as mean ± standard deviation (SD) with normally distributed variables and as median ± interquartile range (IQR) with non-normally distributed values. Instead, categorical data were presented as frequencies (percentages).

Continuous variables were assessed for normality using Shapiro–Wilk test. For normally distributed continuous variables, comparisons between male and female groups were made using the independent Student's t-test. For non-normally distributed continuous variables, the Mann–Whitney *U*-test was employed to compare two groups, while Kruskal–Wallis *H*-test was utilized for comparisons among three groups. For categorical variable, it was evaluated using the chi-squared test for between-group differences.

We first performed a univariate analysis with Spearman's correlation test to evaluate the correlations between the 6MWD and the variables, and then conducted a forward stepwise multivariate linear regression analysis to establish the reference equations for the 6MWD. The most significant categorical variable was added to the model at each step, and the process continued until no additional statistically significant variables were added. Surgery types were coded as 0 = MIDCAB, 1 = OPCAB in regression models. Negative regression coefficients indicate lower 6MWD in OPCAB. A *P* > 0.05 was used to determine whether a variable was entered and removed. Statistical significance is set at *P* < 0.05.

## 3 Results

### 3.1 Demographic characteristics and the 6MWT results

A total of 360 CABG subjects, comprising 285 males and 75 females, who had completed phase Ⅰ CR program and the 6MWT were included in this study. The characteristics and 6MWT results of all participants are presented in Table 1. Females were slightly older than males, with age of 66 ± 8 years compared to 62 ± 13 years (*Z* = −3.864, *P* < 0.001). Males were significantly taller and heavier than females, and differences in BMI were observed between the two genders (25.91 ± 4.11 vs. 25 ± 4.32 kg/m^2^, *Z* = −2.238, *P* = 0.025). The 6MWT was conducted within 5 ± 2 days after CABG surgery, with no significant difference between males and females (*Z* = −1.269, *P* = 0.205).

**Table 1:** Demographic characteristics and 6MWT results of all subjects.

Variables	Male	Female	Total	Statistics	*P*-values
	*n* = 285 (79.2%)	*n* = 75 (20.8%)	*n* = 360 (100%)		
Age, y	62.00 ± 13.00	66.00 ± 8.00	63.00 ± 12.00	Z = −3.864	**<0**.**001**^a^
Type of surgery				*χ*2 = 2.728	0.099^b^
MIDCAB	101 (84.2%)	19 (15.8%)	120 (33.3%)		
OPCAB	184 (76.7%)	56 (23.3%)	240 (66.7%)		
Height, cm	169.16 ± 5.76*	156.68 ± 5.58*	167.00 ± 10.00	t = −16.807	**<0**.**001**^c^
Weight, kg	74.00 ± 14.00	64.00 ± 12.00	71.00 ± 16.00	Z = −8.746	**<0**.**001**^a^
BMI, kg/m^2^	25.91 ± 4.11	25.00 ± 4.32	25.87 ± 3.05*	Z = −2.238	**0**.**025****^a^**
HRi, bpm	89.81 ± 13.76*	88.15 ± 13.20*	89.47 ± 13.64*	t = −0.942	0.347^c^
HRf, bpm	96.48 ± 14.98*	93.12 ± 14.41*	95.78 ± 14.91*	t = −1.742	0.082^c^
HRm, bpm	100.00 ± 21.00	97.00 ± 22.00	99.00 ± 20.00	Z = −1.309	0.190^a^
SBPi, mmHg	126.60 ± 16.65*	129.21 ± 15.23*	127.14 ± 16.38*	t = 1.230	0.219^c^
DBPi, mmHg	75.91 ± 10.03*	75.28 ± 9.49*	75.78 ± 9.91*	t = −0.491	0.624^c^
SBPf, mmHg	132.67 ± 17.86*	132.96 ± 17.26*	132.73 ± 17.71*	t = −0.127	0.899^c^
DBPf, mmHg	77.24 ± 9.88*	74.29 ± 11.26*	77.00 ± 13.00	t = −2.229	**0**.**026****^c^**
SpO_2_i, %	95.00 ± 3.00	95.00 ± 3.00	95.00 ± 3.00	Z = −1.333	0.183^a^
SpO_2_f, %	95.00 ± 3.00	95.00 ± 3.00	95.00 ± 3.00	Z = −1.325	0.185^a^
SpO_2_m, %	94.00 ± 4.00	94.00 ± 4.00	94.00 ± 4.00	Z = −0.172	0.863^a^
RRi, *n*	22.00 ± 4.00	21.00 ± 5.00	22.00 ± 4.00	Z = −0.866	0.387^a^
RRf, *n*	24.00 ± 5.00	23.00 ± 4.00	23.00 ± 5.00	Z = −1.571	0.116^a^
RRm, *n*	24.00 ± 6.00	23.00 ± 6.00	24.00 ± 6.00	Z = −0.898	0.369^a^
Time from surgery to the 6MWT, d	5.00 ± 2.00	5.00 ± 2.00	5.00 ± 2.00	Z = −1.269	0.205^a^
6MWD, m	200.00 ± 100.00	150.00 ± 84.00	183.00 ± 125.00	Z = −5.919	**<0**.**001**^a^
6MWD (% predicted), %	34.00 ± 21.00	29.00 ± 17.00	33.00 ± 20.00	Z = −3.444	**0**.**001**^a^
METs, kcal/kg/h	2.70 ± 0.70	2.40 ± 0.50	2.60 ± 0.90	Z = −5.555	**<0**.**001**^a^

Results expressed as the median ± interquartile range (IQR) or number (%). Bold indicates a statistical difference.

MIDCAB, minimally invasive direct coronary artery bypass; OPCAB, off-pump coronary artery bypass; BMI, body mass index; i, initial; f, final; m, mean; HR, heart rate; SBP, systolic blood pressure; DBP, diastolic blood pressure; SpO_2_, peripheral oxygen saturation; SpO_2_i, resting SpO_2_; SpO_2_f, end-test SpO_2_; SpO_2_m, mean SpO_2_; RR, respiratory rate; 6MWT, 6 min walk test; 6MWD, 6 min walk distance; METs, metabolic equivalent.

^a^ Based on Mann–Whitney *U*-test.

^b^ Based on Chi-square test.

^c^ Based on Independent Student's *t*-test.

* Mean and standard deviation.

The median 6MWD for all subjects was 183 ± 125 meters, corresponding to 33 ± 20% of the predicted value, calculated according to the regression equation derived from healthy subjects (14). The absolute distance walked was positively correlated with gender (*r* = 0.312, *P* < 0.001) and was significantly shorter in females than in males (150 ± 84 meters, 29 ± 17% predicted vs. 200 ± 100 meters, 34 ± 21% predicted, respectively, *Z* = −5.919, *P* < 0.001 for distance; *Z* = −3.444, *P* = 0.001 for percentage of predicted value). Additionally, a significant difference in DBP at the end of the 6MWT was observed between males and females (77.24 ± 9.88 vs. 74.29 ± 11.26 mmHg, *t* = −2.229, *P* = 0.026).

### 3.2 Percentage distribution of the 6MWD

**Figure 1:**
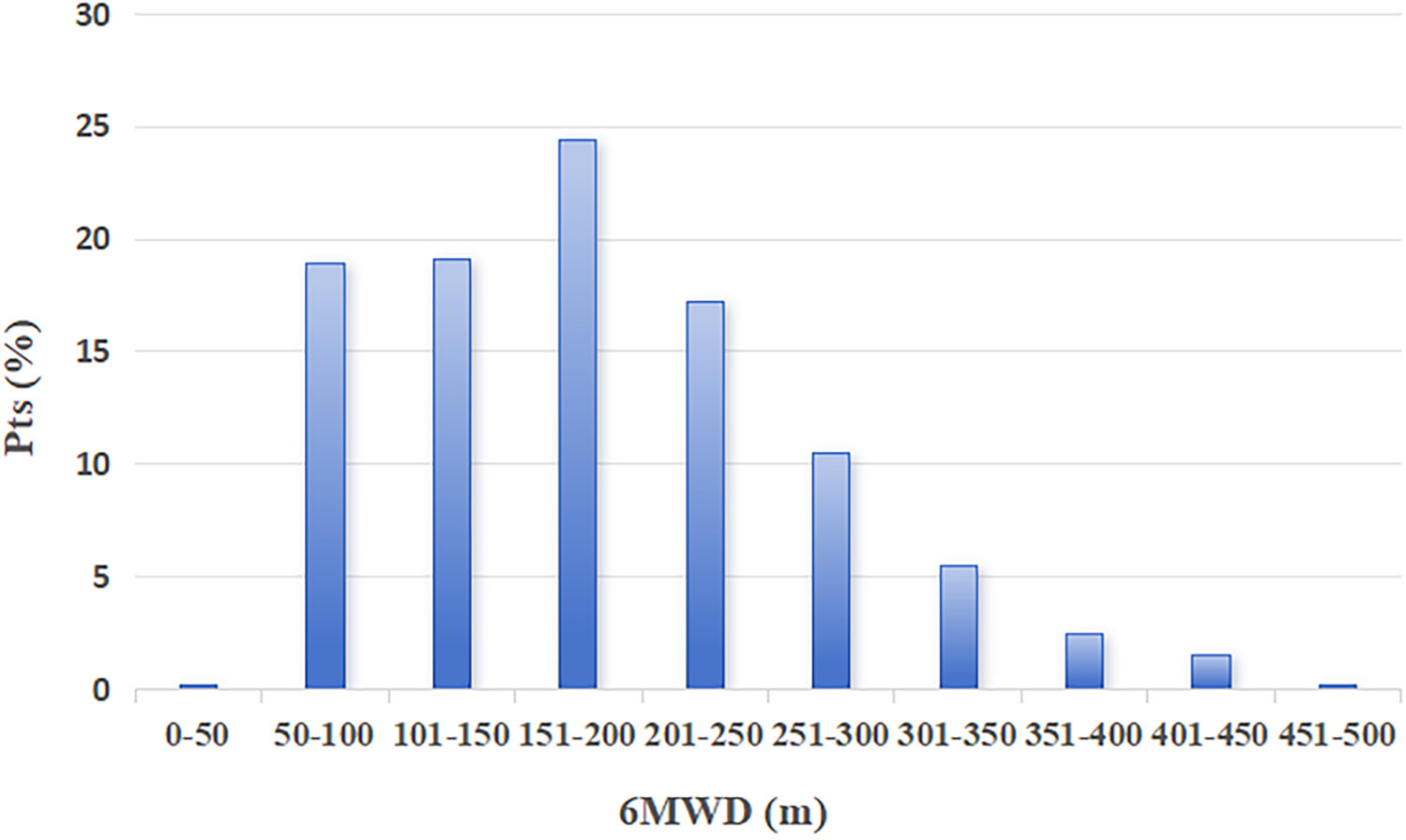
6MWD distribution of all 360 participants.

Figure 1 shows the percentage distribution of 360 patients based on the distance walked after CABG surgery, after completing the phase Ⅰ CR program and before discharge. Notably, approximately three-fifths of these patients exhibited a relevant reduction of exercise tolerance, as indicated by a distance walked ≤ 200 meters.

### 3.3 Associations with the 6MWD

The absolute walking distance was inversely correlated with both the type of surgery (*r* = −0.23, *p* < 0.001) and age (*r* = −0.23, *p* < 0.001) Moreover, in all age groups, males consistently walked significantly greater distances than females, as detailed in Table 2. Furthermore, the absolute distance walked in the 6MWT was lower in patients who were over 75 years of age and had undergone OPCAB surgery (Table 3).

**Table 2:** 6MWD in male and female divided according to age.

Age groups	<65 years	65–75 years	>75 years	Statistics	*P*-values
Male					
N	173	99	13		
6MWD	200 ± 113	179 ± 135	174 ± 186	H = 12.611	**0**.**002**
6MWD (% predicted)	34 ± 19	33 ± 24	36 ± 39		
Female					
N	33	35	6		
6MWD	150 ± 100	143 ± 75	100 ± 113	H = 2.228	0.328
6MWD (% predicted)	30 ± 18	24 ± 16	24 ± 27		

6MWD, 6 min walk distance.

Results expressed as the median ± interquartile range (IQR).

Based on Kruskal–Wallis *H*-test.

Bold indicates a statistical difference.

**Table 3:** 6MWD in 360 CABG patients divided according to gender, age, and type of surgery.

Variables	Meters	% predicted	Statistics	*P*-values
Male (*n* = 285)	200 ± 100	34 ± 21	Z = −5.919	**<0**.**001**^a^
Female (*n* = 75)	150 ± 84	29 ± 17		
Age < 65 years (*n* = 206)	200 ± 101	34 ± 19	H = 20.489	**<0**.**001**^b^
Age 65–75 years (*n* = 134)	165.25 ± 103	33 ± 20		
Age > 75 years (*n* = 19)	150 ± 108	35 ± 28		
MIDCAB (*n* = 120)	200 ± 134	35 ± 23	Z = −3.426	**0**.**001**^a^
OPCAB (*n* = 240)	175 ± 125	33 ± 19		

Results expressed as the median ± interquartile range (IQR). Bold indicates a statistical difference.

MIDCAB, minimally invasive direct coronary artery bypass; OPCAB, off-pump coronary artery bypass.

^a^ Based on Mann–Whitney *U*-test.

^b^ Based on Kruskal–Wallis *H*-test.

The correlations between the 6MWD and various factors are summarized in Table 4. Univariate linear regression analysis revealed that several variables, including age, height, weight, BMI, DBP and SpO_2_ before the test, as well as HR, SBP, DBP, SpO_2_, and RR after the test, and the mean values of HR and RR during the test, were all significantly associated with the 6MWD (all *P* < 0.05).

**Table 4:** Univariate correlation coefficients for the 6MWD and subject variables.

Variables	*r*-values	*P*-values
Age, y	−0.230	**<0**.**001**
Height, cm	0.249	**<0**.**001**
Weight, kg	0.260	**<0**.**001**
BMI, kg/m^2^	0.137	**0**.**009**
HRi, bpm	0.078	0.140
HRf, bpm	0.213	**<0**.**001**
HRm, bpm	0.158	**0**.**003**
SBPi, mmHg	−0.004	0.946
DBPi, mmHg	0.159	**0**.**002**
SBPf, mmHg	0.136	**0**.**010**
DBPf, mmHg	0.173	**0**.**001**
SpO_2_i, %	0.152	**0**.**004**
SpO_2_f, %	0.147	**0**.**005**
SpO_2_m, %	0.100	0.057
RRi, *n*	0.063	0.234
RRf, *n*	0.191	**<0**.**001**
RRm, *n*	0.153	**0**.**004**
Time from surgery to the 6MWT, d	−0.037	0.483

Based on Spearman correlation coefficient. Bold indicates a statistical difference.

BMI, body mass index; i, initial; f, final; m, mean; HR, heart rate; SBP, systolic blood pressure; DBP, diastolic blood pressure; SpO_2_, peripheral oxygen saturation; SpO_2_i, resting SpO_2_; SpO_2_f, end-test SpO_2_; SpO_2_m, mean SpO_2_; RR, respiratory rate; 6MWT, 6 min walk test.

At the stepwise multivariate regression analysis, the 6MWD was positively correlated with gender (*P* < 0.001), HR at the end of the test (*P* = 0.001), and DBP before the test (*P* = 0.043). Conversely, the 6MWD was negatively correlated with the RR at the end of the test (*P* = 0.004). The 6MWD was negatively correlated with OPCAB surgery (*β* = −0.203, *P* < 0.001), indicating shorter distances compared to MIDCAB (Table 5). These factors were identified as independent predictors influencing the 6MWD and explained approximately 18% of the variance in walking distance.

**Table 5:** Multiple regression analysis of the factors for 6MWT.

Variables	Unstandardized coefficients (B)	Standardized coefficients (*β*)	SE	*P*-values
Constant	11.157		36.950	0.763
Gender	53.722	0.256	10.146	**<0**.**001**
Type of surgery	−36.641	−0.203	8.721	**<0**.**001**
HRf, bpm	5.513	0.964	1.615	**0**.**001**
DBPi, mmHg	0.878	0.102	0.431	**0**.**043**
RRf, n	−18.674	−0.812	6.474	**0**.**004**
R square	0.187			
Change in R square	0.176			

Based on a forward stepwise multivariate linear regression analysis. Bold indicates a statistical difference.

SE, standard error; i, initial; f, final; HR, heart rate; DBP, diastolic blood pressure; RR, respiratory rate.

## 4 Discussion

To our knowledge, this study represents the first effort to predict early 6MWD in patients following CABG surgery during phase Ⅰ CR program. Our findings reveal that the physical performance, as assessed by the distance walked during the 6MWT, is significantly reduced in patients who underwent OPCAB surgery, older patients, and females patients following CABG surgery. Notably, the predictors of 6MWD were observed independently of gender, type of surgery, HR and RR at the end of the test, and DBP before the test.

Previous studies have demonstrated that the results of the 6MWT correlate well with changes in symptoms, suggesting that it may serve as supportive evidence for clinical evaluations (16, 17). The distance walked during the 6MWT could be used to predict all-cause mortality in patients who undergo cardiac surgery and to identify high-risk cardiac surgery patients to optimise their post-acute hospital discharge care (18). Our study confirmed the feasibility and safety of conducting the 6MWT in the early postoperative period following CABG surgery. As an observational study conducted in a single CR center, our research reducing the variability of the test performance observed in multimember studies. The results of the 6MWT were reported both as absolute values in meters and as a percentage of the predicted value, based on the reference equation previously published (8). It is important to note that presenting the distance walked in these two distinct formats (absolute value and percentage of predicted value) may have significant clinical relevance.

Our data confirm previous findings that the 6MWD is significant lower in females and elderly patients (19). This may be attributed to the fact that males generally have greater height, higher levels of physical activity, and larger muscle mass compared to females. Additionally, age was negatively correlated with 6MWD, likely due to the gradual decrease in muscle mass, muscle strength, and maximum oxygen uptake that occurs with advancing age. Height, weight, and BMI were positively correlated with 6MWD, probably because taller individuals usually have longer stride lengths, heavier individuals often have greater muscle mass and strength, and a higher BMI may indicate better overall body development and muscle mass, all of which can contribute to increased walking distance. Furthermore, we observed that the 6MWD was significantly shorter after OPCAB surgery compared to MIDCAB surgery, which due to the complexity of cardiac surgery procedure (4, 20). To the best of our knowledge, there are no published reports on the effects of inpatient CR on 6MWT performance in patients who underwent OPCAB versus MIDCAB surgery. We hypothesize that several factors, including reduced surgical trauma, fewer bridging vessels, and shorter surgery time, may have contributed to this difference (3).

In the present study, HR, BP, SpO_2_, and RR measured after the 6MWT were significantly positively correlated with the 6MWD, potentially because these physiological indicators collectively reflect the body's overall capacity to respond to exercise-induced stress. Besides, SpO_2_ and DBP measured before the test were also significantly positively correlated with the 6MWD, which was probably because these metrics provided insights into the patient's baseline cardiorespiratory function and circulatory support capacity. Higher resting SpO_2_ and DBP suggest that patients have better oxygenation and circulatory function prior to exercise, which in turn enables them to sustain longer walking distances during the test (21).

Our findings demonstrate that early 6MWT performance (median 183 meters, 33% predicted) reflects clinically significant physical impairment post-CABG. Crucially, the test's feasibility is supported by: (1) 0% test termination due to adverse events (SpO₂ < 85%, arrhythmia, or symptoms); (2) completion by all 360 patients within 5 ± 2 days post-surgery; and (3) identification of modifiable predictors (e.g., postoperative HR/RR). These results validate 6MWT as a safe, practical tool for quantifying functional capacity during inpatient CR. Early rehabilitation implemented resulted in significant improvements in functional independence post-CABG, which was similar to the findings in Han P et al.'s study and Ohbe H et al.'s study (22, 23). We recommend its integration into phase I CR protocols to guide individualized exercise prescriptions for phase II rehabilitation, particularly targeting high-risk subgroups (females, elderly, OPCAB patients) with markedly reduced 6MWD.

There are several limitations in this study that warrant consideration. First, we did not account for the potential impact of medical therapy on the distance walked at hospital discharge. However, given that exercise tolerance was assessed at the end of a relatively short in-hospital CR program (5 ± 2 days), it is unlikely that medical therapy optimization would have significantly influenced the results. Second, although we collected data on physiological variables at peak exercise during the 6MWT, as well as symptoms and the number of exercise interruptions, these measures were not formally analyzed. This is because they were primarily obtained for patient monitoring and safety purposes rather than for detailed statistical analysis. Third, the 6MWD was expressed as absolute distance (meters) and percentage of predicted values using Enright's equation (14). This equation was derived from healthy populations.While it's widely used, we acknowledge the limitations of making comparisons with healthy populations. Bumrungkittikul et al. reported the independent predictors and equation of 6MWT in post-cardiac surgery, but they predict 6MWT at 4–6 weeks after hospital discharge (24). There is no universally applied equation for the predictive value of 6MWT with reference to post-CABG patients during phase Ⅰ CR program. Results are thus reported as absolute distance and % predicted to contextualize impairment severity. Finally, due to time and staff constraints, we conducted the 6MWT only once for each patient. Previous studies have demonstrated a learning effect in the initial three sessions of the 6MWT, suggesting that a minimum of 2–3 tests should ideally be performed to establish a reliable baseline (25–27).

In conclusion, the early 6MWD in patients following CABG surgery during phase Ⅰ CR program was described for the first time. Our results demonstrate that the 6MWT is feasible and well-tolerated in adult and older patients shortly after CABG surgery. Additionally, inpatient phase Ⅰ CR program following CABG surgery are important determinants of physical functional capacity outcomes. Our study supports the use of the 6MWT as a valuable tool for assessing physical functional capacity during inpatient rehabilitation. Furthermore, we provide useful reference for factors affecting walking distance after CABG surgery, which may be used in the clinical assessment of patients early after CABG surgery and in the development of phase II CR program following hospital discharge. Given these results, further research to investigate interventions that can optimize gains in the 6MWT performance after CABG surgery is warranted.

## Data Availability

The original contributions presented in the study are included in the article/Supplementary Material, further inquiries can be directed to the corresponding author.
